# Thoughts on the end of life in patients with oxygen‐dependent chronic obstructive pulmonary disease: A qualitative interview study

**DOI:** 10.1002/nop2.1463

**Published:** 2022-11-05

**Authors:** Lisa Skär, Christel Borg, Margareta Emtner, Magnus Ekström

**Affiliations:** ^1^ Department of Health Blekinge Institute of Technology Karlskrona Sweden; ^2^ Department of Medical Sciences, Respiratory‐, allergy‐ and sleep research Uppsala University Uppsala Sweden; ^3^ Faculty of Medicine, Department of Clinical Sciences Lund, Respiratory Medicine and Allergology Lund University Lund Sweden

## Abstract

**Aim:**

The aim of the study was to deepen the current knowledge of how patients with chronic obstructive pulmonary disease and long‐term oxygen treatment think about and expect end‐of‐life.

**Design:**

A qualitative design was used.

**Methods:**

A purposeful sample of 19 patients with oxygen‐dependent chronic obstructive pulmonary disease was obtained from the Swedish National Registry on Respiratory Failure (Swedevox). Data was collected with semi‐structured interviews and analysed using a hermeneutic approach.

**Results:**

The analysis revealed three themes: Living in the present without a future; difficulty talking about the uncertainty; and feeling anxious about leaving family behind. Participants indicated that healthcare professionals should invite them to mutual discussions as it was easier to reject an invitation if they could not talk right then, than to initiate a discussion themselves. Start of home oxygen or a deteriorating health status may be an important time to clinically address existential and end‐of‐life issues.

## 1 INTRODUCTION

Chronic obstructive pulmonary disease (COPD) is the fourth leading cause of morbidity and mortality worldwide (Bousquet et al., 2007; Global Initiative for Chronic Obstructive Lung Disease, 2022). COPD is an often progressive and complex illness characterized by chronic airflow limitation, systemic manifestations, and a high burden of comorbidity (GOLD, 2022). The main treatment target in COPD is relief of symptoms, including fatigue, cough, and breathlessness (Barnett, 2005; Vogelmeier et al., 2020). The symptom that patients often experience as most troublesome and limiting is breathlessness (Bowden et al., 2011; Sandberg et al., 2019). COPD is likely to progress over several years and include sudden exacerbations, making the disease unpredictable due to experiences of both good and bad days. Respiratory symptoms may vary in severity over hours, days, and weeks, but are generally worse in the mornings (Miravitlles et al., 2017). Good days are experienced when the breathing is easier, followed by bad days with severe breathlessness. This leads to patients living with uncertainty until the time of death, suffering periods of unpredictable exacerbations with uncertain outcomes (Ek et al., 2015).

Patients with advanced COPD may develop chronic severe hypoxemia, requiring long‐term oxygen‐therapy (LTOT) (Hardinge et al., 2015; Jacobs et al., 2020). Although LTOT improves survival in chronic severe hypoxemia, patients with LTOT still have a high mortality rate, with a median survival time after starting the treatment of <2 years (Ahmadi, Sundh, et al., 2016; Ahmadi, Wysham, et al., 2016). Further, LTOT is often associated with limitations in daily life related to a complex life situation including both physical and psychosocial challenges, such as a “life‐and‐death struggle” connected to anxiety and fear of suffocation and death (Ek et al., 2015; Hardinge et al., 2015; Jacobs et al., 2020). The disease is thereby also perceived as a threat to the quality of life (Long et al., 2019; Zhu et al., 2018). Many patients with COPD lack information about and access to discussions with healthcare professionals on end‐of‐life care issues (Gainza Miranda et al., 2016; Lowey et al., 2013; Pinnock et al., 2011) and many have limited access to palliative care (Maddocks et al., 2017). Furthermore, patients often lack palliative support from healthcare specialists due to it being difficult to determine the prognosis and ascertain when their condition and care moves into the palliative stage (Braço Forte & Sousa, 2017). While the uncertainty in combination with symptoms that are difficult to handle remind people with COPD about death, a sense of engagement at the end of life and a belief in a meaningful life energize patients to better cope with the situation (Ekström et al., 2017).

For people with COPD, the severe symptom burden impacts everyday life when it comes to accepting the life situation because the disease state lacks a clear beginning and has an unpredictable and unexpected end (Zhu et al., 2018). Knowledge about how patients with severe oxygen‐dependent COPD perceive and think about existential and end‐of‐life issues is therefore important as palliative‐care decisions should be individualized based on communication and decision‐making between patients and healthcare professionals (Lowey et al., 2013). The need for improved contact and patient education was highlighted in the recent LTOT guidelines by the American Thoracic Society (ATS) (Jacobs et al., 2020). In addition, the start of LTOT has been suggested as a potential “milestone” in the disease trajectory for identifying and discussing end‐of‐life and palliative‐care needs, to improve outcomes and relieve suffering in this frail patient category (Zhu et al., 2018). Healthcare professionals needs therefore better to understand what these patients' real palliative and care needs are, to conduct care more efficiently at end of life (Gainza Miranda et al., 2016). Therefore, the aim was to deepen current knowledge of how patients with COPD and LTOT think about and expect end‐of‐life.

## 2 METHODS

A hermeneutic approach, described by Fleming et al. (2003), and inspired by Gadamer (1994), was used to elucidate the thoughts and expectations of end‐of‐life of patients with COPD and LTOT. The method is suitable for interviews when the aim is to understand and interpret descriptions of the phenomenon under study. According to Gadamer (1994), understanding starts with a question as it opens different horizons of understanding i.e., an interrelationship between understanding and questioning according to the process of hermeneutic research (Fleming et al., 2003).

### 2.1 Participants

A purposeful sample of 19 participants (7 males and 12 females) was obtained from the Swedish National Registry for Respiratory Failure (Swedevox) at two centres located in south and mid Sweden. Inclusion criteria was persons that had started LTOT for COPD between 2014 and 2016 and living in their own homes. Potential participants in the study were identified by the responsible LTOT nurse; they were informed about the study and were asked if they were interested in participating. The 19 who consented to participate received a letter each containing information about the study, including an informed consent form. The LTOT nurse later contacted the participants willing to participate and scheduled appointments for their interviews. Table 1 lists the characteristics of the participants.

**TABLE 1:** Descriptions of the participants in the study

*n*	Male	Female	Total
Age (mean)	73.3 (4.7)	74.8 (2.9)	74.3 (3.6)
Marital status			
Not married or living alone	‐	1	1
Married, registered partner or domestic partner	6	8	14
Separated	‐	1	1
Widow/widower	1	2	3
Smoker			
Never	1	‐	1
Former smoker	18	7	11
Duration of oxygen therapy			
<6 months	2	3	5
6–12 months	2	2	4
>1 year	3	7	10
Missing	1	‐	1

### 2.2 Ethical considerations

This study was conducted according to the principles of the Declaration of Helsinki (World Medical Association, 2001). The study was approved by the Regional Ethics Review Board (Dnr.2014/528). Informed written consent was provided by all participants. The participants were guaranteed confidentiality and an anonymous presentation of the findings. They were also informed that their participation was voluntary and that they could withdraw from the study at any time, without explanation.

### 2.3 Data collection

In accordance with Fleming et al. (2003) data were collected through semi‐structured qualitative interviews (c.f., DiCicco‐Bloom & Crabtree, 2006). The participants were asked to narrate their experiences about the end of life affected by oxygen‐dependent COPD. The interviews focused on the three areas: (1) experiences of information received about the oxygen treatment, (2) experiences of decision‐making about care at the end of life, and (3) and experiences about support from family members at end of life. When needed, follow‐up and clarifying questions were asked, such as “What happened next?” and “How did you feel then?” A research nurse conducted 10 interviews in one region in the south of Sweden and one of the authors conducted 9 interviews in one region in mid Sweden. The interviews took place in the participants' homes. During some interviews, a relative was present to support the participant. Two pilot interviews were conducted to evaluate the interview areas and structure, which were found to be valid and contain rich information in relation the study aim. The interview areas and structure were unchanged; therefore, the pilot interviews were included in the analysis. The interviews lasted between 20 and 50 minutes. They were audio recorded and transcribed verbatim.

### 2.4 Data analysis

The interviews were analysed following a hermeneutic approach inspired by Gadamer (1994). First, the interview transcripts were read through several times to obtain a sense of the whole. The aim of the study and the researcher's pre‐understanding were central to the reading. Meaning units were then identified in the text for interpretation. Selected meaning units were condensed and compared and, thereafter, arranged in themes based on similarities and differences. This was performed independently by the first and second authors. The themes were then related to the interpretation of the whole text to increase the understanding of the content. The analysis was also discussed with the last author and finally the whole research team reviewed the final article together.

## 3 RESULTS

The analysis revealed three underlying themes:

Living in the present without a future; difficulty talking about the uncertainty; and feeling anxious about leaving family behind. The themes are described below with quotes from the participants. (referred to as P1–19)

### 3.1 Theme: Living in the present without a future

The participants stated that they lived with a fear of planning for the future as they did not know how their illness would progress. They stated that during disease exacerbations, it was difficult to think about the future as they felt that this might be the beginning of the end and that they might not get better again. In connection with receiving LTOT, some had been told that they could survive for a couple of more years, while others were not informed about the prognosis at all. One participant stated;

No one can say how long I will live like this—it's hard not to know. (P12)

The uncertainty of the illness progression in combination with the constant need for oxygen treatment was challenging to handle, contributing to anxiety. Continuous anxiety resulted in the participants choosing to live in the present since it was difficult to think ahead and plan for the future. One participant said;

You are so affected by not knowing how it (the illness) will be so you even cannot think about the future. (P1)

Some participants were in need of informal care and received help from family members. In situations where the participants needed help, their actual state of health became more apparent to them. This caused participants to experience an increased vulnerability that reminded them of their mortality. When friends in their social network passed away, it became another reminder of their own impending death;

When someone close to you dies, you are reminded of your own vulnerability and that it's not far off. (P8)

Some of the participants were also of the view that the future was now and today. Being in the present helped them avoid worries, such as moving to institutional care or no longer being able to take care of themselves;

It's difficult to think about the future as you do not know anything about it and you do not know if the illness will be worsened. (P5)

### 3.2 Theme: Difficulty talking about the uncertainty

The participants stated that they had to adjust their lives around the illness, due to both breathing difficulties and the oxygen treatment itself. Even if they had had the disease for a long time, it was difficult to talk about it and understand the unpredictability of the illness. Worse days in combination with exacerbations were also connected with worsened moods and feelings of sadness and frustration, which also reminded participants that the end was near. They wanted to be invited to discussions about decision making, treatment, and care as it would give them more insight into how their disease progressed over time. The participants requested mutual discussions about the final stage of the disease, but it was difficult to know when this stage was approaching or if they were already there. Further, the participants stated that it should be the healthcare professional's responsibility to contact them and invite them to mutual discussions as it was easier to reject an invitation if they could not talk right then, than to initiate a discussion themselves;

The professionals should call the patients and talk about the end of life, but they did not. (P9)

Participants were frustrated not getting the opportunity to talk about end‐of‐life issues with professionals. They considered it important, but difficult, to discuss how it would be at the end of life, what they could expect, and how they would plan when death approached;

I want to talk about the death, how it will be so I might be prepared. (P3)

The participants explained that they also wanted their loved ones to talk to healthcare professionals so both understood how serious their situation was. The participants found it challenging to explain their dire situation to relatives on their own, and therefore needed support from professionals in having these discussions. Individual discussions were further pointed out as valuable by the participants as they could discuss difficult issues with their relatives without worrying one another.

Further, the participants considered it important to have someone just listen to their thoughts without always needing to find solutions to the problems. They appreciated when the nurse or physician took a little extra time to sit down and talk about their situation. The fact that someone listened made the participants feel much better, and the unpredictability of their situation became easier to handle. The participants further stated that healthcare professionals should be better at listening;

I want to be seen as a human … talking does not cost much, healthcare professionals need to become better at listening. (P7)

### 3.3 Theme: Feeling anxious about leaving family behind

The participants felt responsible for their relatives and found it difficult to acknowledge that they would leave them behind. Some participants also stated that the idea of leaving family behind was more difficult to handle than their impending death. However, all participants expressed that they wanted to die peacefully with their family around. A wish was to fall asleep and not wake up again, preferably at home. It was a security to be with the family, which helped to reduce the fear of death. For participants without close relatives, loneliness was challenging, and some explained that their worst‐case scenario was dying alone. They asked for someone to keep an eye on them so they did not have to be alone, struggling with thoughts about death;

I do not want to be alone when the moment comes, I hope my family will be there. (P10)

At the same time, participants also expressed concern regarding the challenge of being cared for at home and of being a burden to their relatives. Therefore, some of the participants desired to be offered institutional care in the end or that home healthcare services visit them more often, including at night time. Their biggest fear was being affected by breathlessness during the night, which was a situation strongly connected with anxiety.

## 4 DISCUSSION

### 4.1 Discussion of main findings

The main findings of this study were that patients with severe oxygen‐dependent COPD wanted more information and qualified discussions about advanced care planning including the prognosis, treatment options, and what dying might be like. Explanations and information about an illness may validate a patient's experiences, while a lack of explanations negatively influences experiences of being ill (Attre, 2001). When patients understand, the information being given to them about their health condition, it might be easier to ask questions about care and participate in decision‐making for treatment or care (Jangland et al., 2009). However, for the participants in this study, living with severe oxygen‐dependent COPD meant managing severe symptoms that caused major disruption to normal life and handling thoughts about the end of life. The findings show that close relatives were of great importance not only as informal caregivers, but also in encounters with caregivers because they could act as a spokesperson, ask the difficult questions, and be the link to healthcare. This is consistent with Gustafsson et al. (2013), notion of involving close relatives in relationships with healthcare professionals as it brings coherence in difficult situations. On the contrary, close relatives who have the opportunity to develop a relationship and have continuity in encounters with caregivers are afforded the opportunity to support the patient (Eriksson, 2006). When close relatives and healthcare professionals have established a relationship, the opportunity to share important information about the patient increases, helping the patient to better cope with the difficult situation (Nygren Zotterman et al., 2018).

Previous research about patients with COPD experiences in the end‐of‐life phase has similarities to the findings in this study. The findings indicate that insufficient communication on the prognosis compounded the uncertainty among patients and their healthcare professionals regarding the likely illness trajectory, leading to difficulties in thinking and planning ahead. This was expressed as leading to feelings of anxiety and frustration and to participants choosing to rather live in the present, which could be viewed as a coping strategy to avoid worries related to the potential need for institutional care or no longer being able to take care of themselves. Carlucci et al. (2012) state that patients with COPD often have unmet information and communication needs. Experiences of insufficient communication may reflect patients' need for information about the nature and course of COPD, such as results of disease progression, pulmonary function, and therapeutic plans (Nelson & Hamilton, 2007). Research (Lari et al., 2014), highlights the importance of communication between patients with COPD and their healthcare professionals and ascribes it as a vital element in the care process since good communication is required for effective management of COPD (Lowey et al., 2013). Ensuring good communication includes giving the patient enough time and ensuring adequate opportunities for the patient, close relatives, and caregivers to ask each other questions (Nygren Zotterman et al., 2016). This is in line with the person‐centred approach, which suggests that care should be based on trust and confirmation and a shared understanding based on a partnership (McCormack & McCance, 2016). Patient's experiences and a care plan could thereby be developed based on a mutual understanding. Care based on a shared understanding has been shown to have a significant impact on both the patient–caregiver relationship as well as the patient's health and wellbeing outcomes (Gardiner et al., 2009). When patients do not understand the information being shared, they may find it difficult to participate in the decision‐making and care planning (Petronio & Di, 2012).

Our findings show that the participants felt anxious about struggling with thoughts about death; with a specified worst‐case scenario being dying alone. While the participants wanted to die peacefully with their family around, they also expressed conflicting feelings of being a burden to their relatives and sadness from the thought of leaving loved ones behind. Research (Pinnock et al., 2011) has shown that people with severe COPD have anxieties regarding the end of life, death, and dying. The complexity about the end of life requires a detailed care planning of necessary healthcare, including support and care to relieve symptoms and anxiety (Gardiner et al., 2009). Limited abilities thereby often become a struggle between waiting to die and being able to handle the end‐of‐life situation (Ekdahl et al., 2021). A caring team including different professionals, such as nurses, physicians, and close relatives, plays a vital role in ensuring that COPD patient‐care improves. Patients with COPD should, in the end‐of‐life phase, be considered with the aim of recognizing all clinically evident symptoms, treating them in the best way possible and continuously discussing their wishes about end‐of‐life treatment (Carlucci et al., 2012).

Research on COPD patients with the ability to manage their end‐of‐life phase has similarities to the findings of this study. Gardiner et al. (2009) state that limited abilities result in a struggle between waiting for the end and being able to handle the anxiety resulting from this complex situation. To stabilize the situation, detailed planning is required to manage everyday life, which may improve patients', their relatives', and caregivers' insights into the likely disease progression and patient needs over time (Ekdahl et al., 2021). The participants in this study particularly stressed that it should be the healthcare professionals responsibility to raise issues and initiate a discussion as to how patients should manage daily life and their anxiety about death. The participants felt they needed support to discuss these difficult issues with their healthcare professionals, without worrying their close relatives. Management models with active disease‐modifying treatments combined with palliative‐care interventions with a shared decision‐making show great results for good‐quality end‐of‐life care for patients with COPD (Spathis & Booth, 2008). Discussions should be tailored to the patient's situation and needs, including their health status, social situation, values, and expectations for the time ahead and for the end of life. Start of home oxygen or a deteriorating health status may be an important time to clinically address existential and end‐of‐life issues. This aligns with the person‐centred perspective that the team of caregivers needs to be on hand to support patients deal with their anxiety about death through advance care planning (McCormack & McCance, 2016).

### 4.2 Strengths, limitations, and trustworthiness

For the study, a purposeful sample of 19 individuals who had experienced the phenomenon under study were invited to participate. The participants were obtained from the Swedish National Registry for Respiratory Failure (Swedevox) located at two centres. As the recruitment was conducted by members of the clinical team it was important that the presumptive participants received an information letter without pressure to participate which was voluntary. The strengths of this study include in‐depth interviews with a relatively large sample of patients with ongoing LTOT. Interviews were conducted by dedicated research staff not involved in care and treatment but with extensive clinical experience of communicating with people with advanced disease and frailty. To ensure the trustworthiness of the results, the researcher is responsible for providing enough details regarding the research process to ensure confirmability, credibility, and truthfulness (Fleming et al., 2003). To reach confirmability, the researchers discussed the analysis during the whole process and returned to the interview texts several times. Quotations from the interviewees were used to enhance the reader's possibility of deciphering the content in the results, which establishes credibility. Based on the researchers' interpretations and understanding as physicians, registered nurses, and researchers, truthfulness was established when an agreement among the parts and whole was achieved. The authors' goal was to present the most probable interpretation based on the analysis.

## 5 CONCLUSION

The findings of this study affirm that patients with COPD and LTOT require qualified discussions with healthcare professionals and that it is their responsibility to initiate mutual discussions about the end of life. End‐of‐life fear in COPD patients are based on not only illness severity, but also on disease‐specific anxieties expressed by patients or close relatives. Start of home oxygen or the deterioration in health status may be an important time to clinically address existential and end‐of‐life issues. When this happens, the patient and close relatives would be prepared about the unpredictability of the end of life and feelings of anxiety could decrease. In the absence of a cure for COPD, advance care planning based on mutual discussions is of utmost significance; this should be based on a partnership between the patient, close relatives, and healthcare professionals, considering the patient's needs and wishes to facilitate a good life and improved quality of life near death.

## AUTHOR CONTRIBUTIONS

Conceptialisation: Magnus Ekström, Margareta Emtner. Formal analysis: Lisa Skär, Christel Borg. Investigation: Margareta Emtner. Methodology: Lisa Skär, Christel Borg, Margareta Emtner. Validation: Lisa Skär, Christel Borg, Margareta Emtner, Magnus Ekström. Writing‐original Draft: Lisa Skär, Magnus Ekström. Writing‐review & editing: Lisa Skär, Christel Borg, Margareta Emtner, Magnus Ekström.

All authors have agreed on the final version and meet at least one of the following criteria [recommended by the ICMJE (http://www.icmje.org/recommendations/)]:

- substantial contributions to conception and design, acquisition of data or analysis and interpretation of data.
- drafting the article or revising it critically for important intellectual content.

## ETICHAL APPROVAL

This empirical study was conducted according to the prinicples of the Declaration of Helsinki and approved by the Regional Ethics Review Board.
